# Fondazione Italiana Linfomi (FIL) expert consensus on the use of intensity-modulated and image-guided radiotherapy for Hodgkin’s lymphoma involving the mediastinum

**DOI:** 10.1186/s13014-020-01504-8

**Published:** 2020-03-12

**Authors:** Andrea Riccardo Filippi, Sofia Meregalli, Anna DI Russo, Mario Levis, Patrizia Ciammella, Michela Buglione, Andrea Emanuele Guerini, Giuseppina De Marco, Vitaliana De Sanctis, Stefano Vagge, Umberto Ricardi, Gabriele Simontacchi, on behalf of the Fondazione Italiana Linfomi (FIL) Radiotherapy Committee

**Affiliations:** 1 Radiation Oncology Department, Fondazione IRCCS Policlinico S. Matteo, Viale Golgi 19, 27100 Pavia, Italy; 2 Azienda Ospedaliera San Gerardo, Monza, Italy; 3 Fondazione IRCCS Policlinico San Matteo and University of Pavia, Viale Golgi 19, 27100 Pavia, Italy; 4 Università di Torino, Torino, Italy; 5 IRCCS Arcispedale S. Maria Nuova, Reggio Emilia, Italy; 6 Università di Brescia, Brescia, Italy; 7 Policlinico di Modena, Modena, Italy; 8 Università La Sapienza, Rome, Italy; 9 IRCCS Policlinico S. Martino, Genoa, Italy; 10 Azienda Ospedaliera Careggi, Florence, Italy

**Keywords:** Lymphoma, Intensity-modulated radiotherapy, Image-guided radiotherapy

## Abstract

**Aim:**

Advances in therapy have resulted in improved cure rates and an increasing number of long-term Hodgkin's lymphoma (HL) survivors. However, radiotherapy (RT)-related late effects are still a significant issue, particularly for younger patients with mediastinal disease (secondary cancers, heart diseases). In many Centers, technological evolution has substantially changed RT planning and delivery. This consensus document aims to analyze the current knowledge of Intensity-Modulated Radiation Therapy (IMRT) and Image-Guided Radiation Therapy (IGRT) for mediastinal HL and formulate practical recommendations based on scientific evidence and expert opinions.

**Methods:**

A dedicated working group was set up within the Fondazione Italiana Linfomi (FIL) Radiotherapy Committee in May 2018. After a first meeting, the group adopted a dedicated platform to share retrieved articles and other material. Two group coordinators redacted a first document draft, that was further discussed and finalized in two subsequent meetings. Topics of interest were: 1) Published data comparing 3D-conformal radiotherapy (3D-CRT) and IMRT 2) dose objectives for the organs at risk 3) IGRT protocols and motion management.

**Results:**

Data review showed that IMRT might allow for an essential reduction in the high-dose regions for all different thoracic OAR. As very few studies included specific dose constraints for lungs and breasts, the low-dose component for these OAR resulted slightly higher with IMRT vs. 3D-CRT, depending on the technique used. We propose a set of dose objectives for the heart, breasts, lungs, and thyroid. The use of IGRT is advised for margin reduction without specific indications, such as the use of breath-holding techniques. An individual approach, including comparative planning and considering different risk factors for late morbidity, is recommended for each patient.

**Conclusions:**

As HL therapy continues to evolve, with an emphasis on treatment reduction, radiation oncologists should use at best all the available tools to minimize the dose to organs at risk and optimize treatment plans. This document provides indications on the use of IMRT/IGRT based on expert consensus, providing a basis for clinical implementation and future development.

## Background

The combination of brief chemotherapy followed by radiation therapy (RT), based on a risk-adapted and response-adapted strategy, is a therapeutic standard for early-stage Hodgkin lymphoma (HL) [1]; nonetheless, the role of radiation is still debated, with some concerns for late toxicity (second malignancies, cardiac disease). Advances in imaging, treatment planning, and RT delivery have made it possible to better define and further reduce RT fields in many situations [2]. Current RT protocols combine limited radiation volumes with advanced planning and delivery techniques, such as intensity-modulated RT (IMRT) and Image-Guided Radiotherapy (IGRT) [2].

Various IMRT solutions have been implemented over the years, generally obtaining superior target coverage and better OAR sparing, mainly heart and coronary arteries, and reducing the lung volumes treated at high-dose (see next paragraph for a comprehensive review). The benefit achievable on heart structures is usually counterbalanced by a more significant proportion of breasts and lungs receiving low-dose (below 5 Gy). Given this unique dose distribution, the appropriateness of IMRT in young HL patients has been questioned, assuming a potential increase in radiation-induced malignancies [3]. Several comparative planning studies for mediastinal lymphomas, including secondary cancer risk modeling, have been conducted over the last decade [4]. Recent modeling studies partially overturned this hypothesis, assuming for low-dose a slight risk of second malignancy induction with fractionated radiotherapy in adults [5, 6]. The second-generation comparative planning studies have further optimized heart sparing, adopting various technical solutions. In studies integrating radiobiological models, the dose distribution on breasts and lungs did not translate in an increased risk of secondary cancer or a reduction of life expectancy [7, 8]. The variable anatomic presentation of early-stage HL may significantly affect second cancer and cardiac risk of HL survivors on an individual basis, a factor that often steers the selection of the appropriate planning solution. Cardiac toxicity further emerged as a critical point, given its linear correlation with mean heart dose [9] and with some non-treatment related risk factors. A few studies have investigated the dose-response relationship of single heart substructures [10, 11], suggesting that the mean heart is probably inadequate for predicting all types of radiation-related heart diseases. However, we still do not have explicit dose constraints on single heart substructures to be implemented in clinical use. Although the advantages of IMRT include the tightly conformal doses and steep gradient next to healthy tissues, target definition and treatment delivery verification need even more attention than with conventional RT to avoid the risk of geographic miss and subsequent decrease in tumor control. IGRT protocols assume an essential role in ensuring full target coverage during the whole treatment.

## Methods

A dedicated working group was set up within the Fondazione Italiana Linfomi (FIL) Radiotherapy Committee in May 2018. After a first meeting, the group adopted a dedicated platform to share retrieved articles and other material. Two group coordinators redacted a first document draft, that was further discussed and finalized in two subsequent meetings. We here report the final document, consisting of a) an extensive review of published literature on modern volumes definition and dosimetric studies comparing 3D-conformal radiotherapy (3D-CRT) and different IMRT solutions b) a proposal for mediastinal organs at risk contouring, individualized risk estimation and dose objectives and c) IGRT protocols and motion management. The final indications were discussed and approved by the expert panel, and represent the opinion of expert radiation oncologists from different Centers affiliated to the Italian FIL network.

## Results

### Modern treatment volumes definition

Modern contouring protocols recommend limited RT volumes, based on the principles of involved node radiation therapy (INRT) and Involved Site Radiation Therapy (ISRT) [12]. Volumes reduction started with the advent of CT simulation and 3D reconstruction software, which laid the groundwork for a new way of considering lymphoma radiation fields in comparison with the 2D era. Baseline imaging (CT and PET-CT), obtained before chemotherapy, then became the basis for target volumes delineation (corresponding to involved lymphatic sites at diagnosis) [2]. This approach is behind the concept of INRT, developed by the EORTC in H10 trial [13] and involved-site radiation therapy (ISRT), ideated by the International Lymphoma Radiation Oncology Group (ILROG) and described in details in dedicated guidelines [14]. In both INRT and ISRT, the pre-chemotherapy disease extension determines the clinical target volume, and the resulting treated volume is significantly smaller than with involved-field radiotherapy (IFRT). INRT represents a particular case of ISRT, in which pre-chemotherapy imaging is obtained in ideal conditions for post-chemotherapy treatment planning (e.g., same position). High-quality retrospective clinical data show that INRT/ISRT is safe and effective in terms of disease control, though the median follow-up time is < 5 years, and the sample size small [15–18]. The definitive results of the H10 prospective trial confirmed INRT safety for both favorable or unfavorable presentations (EORTC criteria). Thus, both INRT and ISRT are now considered as the standard of care for Hodgkin’s Lymphoma patients [1].

### Dosimetric studies of IMRT vs. 3D-CRT

Two members of the expert panel performed a literature review using the keywords “Intensity-Modulated Radiation Therapy” or“ IMRT” or “Volumetric Modulated Arc Therapy” or “VMAT” and “mediastinal (or mediastinum) lymphoma,” without time restriction (last updated August 30th, 2019) across PubMed, EMBASE, and Scopus. They identified a total of 121 records, and a first selection was performed based on the titles and abstracts, including only articles published in English, and excluding duplicates, case reports, reviews without original data and letters to the editor. Publications reporting on IMRT only, proton therapy only, or comparing different volumes (i.e., IFRT vs. INRT or mantle-field) without comparing different techniques were then excluded. The panel finally selected 21 publications, reporting on a comparison between 3D-CRT and IMRT, independently of the IMRT technique used (static fields, volumetric) or the definition of the target volumes (IFRT, INRT or ISRT).

#### Heart, lungs, breasts, thyroid

Table 1 summarizes the main findings of these studies, focusing on the dose distribution on the heart, lungs, breasts, and thyroid [7, 19–38].

**Table 1:** Main dosimetric findings from studies comparing 3D-CRT vs. IMRT

	Heart AP-PA	Heart 3D-CRT	Heart IMRT	LAD AP-PA	LAD 3D-CRT	LAD IMRT	Lung AP-PA	Lung 3D-CRT	Lung IMRT	Breast AP-PA	Breast 3D-CRT	Breast IMRT	Thyroid AP-PA	Thyroid 3D-CRT	Thyroid IMRT
**Goodman et al 2005** [19]**: 11 pts HL and 5 pts NHL; 18–45 Gy (median 36 Gy). IMRT: 3–6 beams**	MD 19.1 Dmax 35.4	MD 19.3 Dmax 36.2	MD 16.5 Dmax 37.2				MD 16.7 V20 51	MD 16.2 V20 68	MD 13.7 V20 64						
**Girinsky et al 2006** [20]**: 12 pts HL with residual disease; 2/40 Gy IMRT: 5 beams, different angles**	MeD 7.8 V30 24.7	MeD 9,1 V30 19.7	MeD 7.7 V30 14	MD 33	MD 32	MD 30	MD 11.2 V20 24.7	MD 14 V20 27.5	MD 12.8 V20 24.5	MeD 0.3 V20 4.8 V5 8	MeD 0.7 V20 4.2 V5 17	MeD 1.2 V20 3.5 V5 26			
**Nieder et al 2007** [21]**: IMRT: 7-field coplanar** **2/30 Gy**	Med 0.1–30; V30 2–46; V7.5 27–94; V4.5 29–96	Med 0.1–18.6; V30 4–20; V7.5 26–93; V4.5 31–96	Med 0.1–12.6; V30 4–9; V7.5 20–67; V4.5 25–80	Med 23.4–28.5; V30 0–90; V7.5 69–100	Med 21.3–28.5; V30 0–23; V7.5 72–100	Med 11.1–15.9; V30 0–3; V7.5 70–88	Right-left: MD 1.5–2.4; V20 24–32; V30 15–20	Right-left MD 16.2–16.2; V20 20–26; V30 8–11	Right-left MD 13.8–13.2; V20 23–27; V30 3–6	Right:MeD 0.9, V25 5, V5 12. Left: MeD 0.9, V25 7, V5 15	Right: MeD 1.5, V25 19, V5 46. Left: MeD 1.5, V25 24, V5 48	Right: MeD 5.1, V25 1, V5 100. Left: MeD 4.8, V25 1, V5 100			
**Chera et al 2009** [22]**: nine stage II HL; INRT 2/30 Gy.** **IMRT: 5 equally spaced beams**							MD 4.8 V4 20.7 V10 15.3 V20 11.2 V30 6.2		MD 5.4 V4 37.5 V10 21.1 V20 6.8 V30 0.8	MD 1.9 V4 7.9 V10 6.5 V24 3.6 V30 0.8		MD 3.7 V4 25.3 V10 16.8 V24 1.6 V30 0.3	MD 7.1 V4 28.2 V10 21 V24 14 V30 5.3		MD 9.1 V4 44.6 V10 34.4 V24 15.9 V30 4.6
**Cella et al 2010** [23]**: 10 pts supra-diaphragmatic HL; 1.5/30 Gy.** **Forward planned IMRT**	MD 21.2 Dmax 32.3		MD 21.8 Dmax 32.4				MD 16.5 V20 45.4 V30 28.5		MD 16.2 V20 45.1 V30 23.4	MD 8.9 V20 21.2 Dmax 34.2		MD 7.4 V20 20.7 Dmax 33	MD 27.7 V30 79 Dmax 33.3		MD 25.3 V30 20.8 Dmax 30.8
**Weber et al** [24]**: 10 early-stage supra-diaphragmatic HD female patients; 2/30 Gy IFRT vs INRT** **IMRT: 9 equally-spaced beams vs Rapidarc**								IFRT-INRT MD 9.1–9.1; V3 46.7–33.3; V10 33.2–21.5	IFRT-INRT MD 8.5/8.3–6.9/ 6.5; V3 67.4/67.7–57.9/ 18.8; V10 34.2/31.4–25.9/ 3.7		IFRT-INRT MD 4.9–2.5; V3 6.6–2.9; V10 3.9–1.5	IFRT-INRT MD 2.9/2.9–1.6/1.5; V3 25.1/24.1–14.2/13.6; V10 9.2/6.6–3.5/2.2		IFRT-INRT MD 22.6–15.2; V3 91.7–67.5; V10 83.5–49.4	IFRT-INRT MD 23.7/23.3–17.5/16.8; V3 90.8/ 91.7–84.1/ 84.3; V10 88.2/85.8–74.3/65.1
**Hoppe et al 2012** [25]**: 12 pts St I-III HL; 1.8/30.6–39.6 Gy. IMRT: 5–7 beams coplanar or non-coplanar**		MD 19.4; V4 79.1;V10 70.5; V20 56.5; V30 38.1	MD 12.2; V4 70.6; V10 47.4; V20 28.1; V30 13.6					MD 13.2; V4 56.2; V10 44.6; V20 34.9; V30 21.6	MD 10.6; V4 65.4; V10 41.4; V20 19.9; V30 3.6		MD 5.4; V4 23.3; V10 18.8; V20 13.3; V30 8.5	MD 5.5; V4 37.4; V10 21.8; V20 8.5; V30 0.8		MD 18.8; V4 77.5 V10 62.4 V20 53.5 V30 25.8	MD 19.3; V4 98.8 V10 78.5 V20 49.8 V30 11
**Koeck et al 2012** [26]**: 20 pts (10 M, 10 F) early mediastinal HD; dose 2/30 Gy. IF vs IN. Coplanar IMRT**		MD 17.9–9.2; V4 74.8–39.5; V10 66.9–32.6, V25 49.6–21.7	MD 13.8–7.4; V4 94.2–50.4, V10 61.7–27.8; V25 12.4–6.7					MD 10.6–8.6; V10 37.7–30.2; V20 27.8–21.9	DM 12.8–9.6; V10 58.5–39.7; V20 16–11.3		DM 4.4–3.4; V4 22.6–17.1; V10 17.1–13; V25 3.8–3.3	DM 6–4.6; V4 61.8–38.6, V10 15.8–13.7, V25 0.8–0.6			
**Campbell et al 2012** [27]**: 10 females, stage I–IIA supra-diaphragmatic HL. INRT 1.8/30.6 Gy. IMRT: VMAT**		MD 6.9; V30 11.1	MD 4.3; V30 1.6		MD 25	MD 24.7		MD 7.3; V5 28.1; V20 18.6	MD 6.9; V5 45.3; V20 8.1		MD 1.6; V5 6.1; V20 3.3	MD 2.1; V5 19.6; V20 0.2		MD 13.5; V4 53.4; V20 37.9	MD 15.5; V4 75; V20 37.7
**De Sanctis et al 2012** [28]**: 10 supra-diaphragmatic St. II HL. IFRT 2/30 Gy. IMRT: 5 equally spaced beams.**		MD 4.3; V5 15.8; V10 12.9; V20 7.6; V30 1.3	MD 4.2; V5 25.7; V10 15.3; V20 6.5; V30 0.2		MD 9.9; V5 44; V10 39.2; V20 25.7; V30 6.2	MD 9; V5 52.8; V10 44.5; V20 23; V30 0.9		MD 6.8; Left-Right: V5 28.9–30.3; V10 20.5–23.2; V20 13.2–14.7; V30 4.7–2.6	MD 8.7; Left-Right: V5 48.1–44.8; V10 30–32.8; V20 7.8–7.9; V30 0.1–0.1		MD 1; Left-Right: V5 4.1–3.3; V10 2.1–1.7; V20 1.1–0.9; V30 0.2–0.8	MD 2.3; Left-Right: V5 20.6–17; V10 13.6–11; V20 0–0; V30 0–0		MD 15.3; V10 64.8; V20 46.4; V30 16.2	MD 21.4; V10 95; V20 54.6; V30 9.6
**Fiandra et al 2012** [29]**: 10 female mediastinal HL patients. INRT 2/30 Gy. IMRT: VMAT vs TD vs HT**		MD 5.1	MD 3.1–4.3		MD 22.1; V20 73.5	MD 15.7–19.8; V20 43.1–59.1		MD 6.6; V5 29.3; V10 22.6; V20 15.2; V30 3.5	MD 5.9–6.4; V5 26.4–39; V10 20.6–25.6; V20 7.6–15.6; V30 0.1–0.7		MD 1 Gy, V4 4.5; V10 2.5; V20 0.9; V30 0.2	MD 0.7–1.2; V4 3.7–6.2; V10 1.1–2.4; V20 0.1–1.3; V30 0–0.6		MD 17.7; V18 45.9; V25 32	MD 13.5–17.3; V18 33.4–51.3; V25 21.3–32.9
**Chen et al 2012** [30]**: 19 pts mediastinal HD or NHL; dose 2/36 Gy.** **IMRT. Coplanar vs non-coplanar**	MD 17; V30 34.5		MD 14.5–15, V30 22.4–22.1				MD 13.3; V5 50, V10 42.5 V20 32.2		MD 13.8–12.9; V5 61.2–53.9; V10 48–43.2; V20 27.9–26.9	MD 2.8; V5 12, V10 8.1, V20 6.4		MD 3.3–2.9; V5 16%, V10 8.4–8, V20 4–4			
**Cella et al 2013** [31]**: three PTV scenarios, supra-diaphragmatic HD. 1.5/30 Gy.** **IMRT: forward, inverse or Tomo**	V25 22.5–60.5		V25 from 21 to 4.5 to 8.7–67.5				V20 12.1–30.7; MD 5.3–11.9		V20 from 15 10.1–15 to 23–28.8; MD from 4.9–8.1 to 11.1–13.5				V30 49–93.9		V30 from 16.9–48 to 45–60
**Antoni et al 2013** [32]**: 13 pts supradiaphragmatic St. II HL. INRT 2/30 Gy + 2/6 Gy boost. IMRT: Helical Tomotherapy**		MD 13; V20 32.2; V30 23.1	MD 11.2; V20 20.7; V30 11.1					MD 12; V5 47.1; V20 28.2; V30 19.4	MD 9.2; V5 56.4; V20 12.2; V30 4.9		MD 2.7; V5 27; V20 5.7; V25 5.1; V30 4.1	MD 4.7; V5 9.9 V20 3.9; V25 1.2; V30 0.2		MD 34.6; V30 87.7	MD 31.6; V30 72.7
**Maraldo et al 2013** [7]**: 27 patients, St I-II supra-diaphragmatic HL. INRT 1.8/30.6 Gy. IMRT: VMAT**		MD 9.9	MD 10.1					MD 8.6	MD 11.4		MD 3.0	MD 7.5			
**Voong et al 2014** [33]**: 9 female pts with mediastinal HL; INRT 1.8/30.6 Gy.** **IMRT: butterfly, multiple arcs**	MD 14.3 V30 29 V20 38.8 V5 46		MD 11.5 V30 16.8 V20 28.3 V5 42.5	MD 14.5 V30 28.5 V20 42.2 V5 51.7		MD 10.4 V30 21.5 V20 28 V5 51.8	MD 11.3 V30 19.5 V20 29.9 V5 41.1		MD 9.3 V30 4.8 V20 22.2 V5 54	Right: MD 1.9, V30 1.9,V20 3.7, V5 7.5. Left: MD 2.4, V30 2.9,V20 4.9, V5 8.4		Right: MD 2.3, V30 0.5, V20 3.8, V5 13.7. Left: MD 2.4, V30 1.2, V20 4, V5 11.2			
**Aznar et al 2015** [34]**: 22 pts early-stage mediastinal HL; INRT 1.8/30.6 Gy free breath vs breath hold; IMRT: different techniques**		FB vs DIBH MD: 8.7–5.4; V20 19.8–14.5	FB vs DIBH MD: 8.1–4.5; V20 15.7–7.9					FB vs DIBH MD: 10.3–8.2; V20 20.8–20.7	FB vs DIBH MD: 9.8–8.2; V20 17.9–13.7		FB vs DIBH MD: 2.3–3.2	FB vs DIBH MD: 4.2–4.8		FB vs DIBH MD: 22.1–25.9	FB vs DIBH MD: 25.4–28.2
**Kriz et al 2015** [35]**: 11 pts mediastinal early HL; 1.8/30.6 Gy INRTvs IFRT, DIBH vs free breath. IMRT: 5 beams vs 7 equally spaced beams.**	MD 4–12.6		MD 4.1–15.4				MD 5.9–9.1; V10 18.3–32.6; V20 14.1–25.4		MD 6.5–13; V10 25.8–57.8; V20 9.5–24.1	Left: MD 2–4.5; Right: 0.6–1.5		Left: MD 2.4–7.4; Right: 1.2–5.5			
**Besson et al 2016** [36]**: 69 pts with HL or NHL; doses calculated for 2/30 Gy. IMRT: Tomotherapy**	MD 11.7 MeD 8.4 V4 45 V20 34.1 V30 16.6		MD 9 MeD 7 V4 51.2 V20 18.6 V30 3.4				MD 9.6 MeD 8.7 V4 42.4 V20 25.6 V30 9.8		MD 9.5 MeD 11.1 V4 62 V20 16.2 V30 2.3						
**Horn et al 2016** [37]**: 14 females with stage II mediastinal HL. ISRT 2/30 Gy. IMRT: Tomotherapy**		MD 11.3; Dmax 30.9; V4 41.8; V10 36.7; V20 31.1; V30 11.8	MD 8.5; Dmax 30.8; V4 46.2; V10 33.8; V20 23.4; V30 10.1					Left-Right MD 9.4–7.5; V4 41.3–33.6; V10 31.7–24.7; V20 25.4–18.6; V30 10.1–6.5	Left-Right MD 8.6–7.8; V4 56.4–55.2; V10 36.2–30.8; V20 13.8–12; V30 0.3–0.7		Left MD 5.6; V4 22.8; V10 18.6; V20 14.6; V30 4.9 Right MD 3.6; V4 14.8	Left MD 4.9; V4 36; V10 19.9; V20 4.2; V30 0.2 Right MD 3.8; V4 33.4			
**Higby et al 2016** [38]**: 11 pts HL st. I-II; ISRT 1.8/30.6–36 Gy.** **IMRT: VMAT**		MD 15.8 V30 24.7	MD 12.8 V30 17.7					MD 13 V20 35.4 V5 52.6	MD 11.9 V20 20.8 V5 67.8		Left:V5 5.3, V10 4.5 Right: V5 4.6, V10 1.3	Left: V5 39.5, V10 17.7 Right: V5 32.4, V10 11.2%.			

*Abbreviations*: *MD* mean dose, *MeD* median dose, *V* volume

#### Integral dose

IMRT usually increased the so-called “low-dose bath” to the rest of the body when compared to the antero-posterior/postero-anterior (AP-PA) approach and 3D-CRT [20, 22, 38]. However, a few techniques, including non-coplanar IMRT [31], allowed for a reduction of the mean integral dose. Conversely, a reduction of the volume exposed to high-dose was repeatedly reported. The dose to the rest of the body in the study by Girinsky et al. [20] was significantly lower with AP-PA and 3D-CRT than with IMRT (the mean dose was 0.4 Gy, 1.3 Gy, and 2.8 Gy, respectively). Chera et al. [22] reported a significant decrease in total body V30 and V24 with IMRT vs. AP-PA. In a comparison of 3D-CRT vs. Helical Tomotherapy (HT), Higby et al. [38] identified an increase of body V5 from 23.3 to 32.7%, while V10 was almost equal (20.6% vs. 20.5%, respectively). Hoppe et al. [25] reported a 22% increase of body V4 for IMRT compared to 3D-CRT, while V10 was similar, and V20 and V30 were reduced by 41 and 63%, as was the mean dose (7.7 Gy vs. 9.5 Gy, respectively). No significative differences were found in terms of integral dose among all techniques (HT, VMAT, Butterfly-VMAT, TomoDirect, and 3D-CRT) in the article by Fiandra et al. [29].

#### PTV coverage with IMRT

IMRT allowed superior or at least equivalent Planning Target Volume (PTV) coverage compared to AP-PA plans or multi-fields 3D-CRT [19, 21–23, 26, 28–30, 32, 33, 35, 36, 38]. Dosimetric parameters relative to PTV coverage, from studies reporting these data, are summarized in Table 2.

**Table 2:** PTV coverage in studies comparing 3D-CRT vs. IMRT

	AP-PA	3D-CRT	IMRT
**Goodman et al** [19]**: IMRT 3–6 beams**	V95 93%;D95 90%; Dmax 117%; Dmin 78%; MD 103%	V95 95%; D95 98%; Dmax 118%; Dmin 80%; MD 106%	V95 98%; D95 101%; Dmax 120%; Dmin 76% MD 107%
**Nieder et al** [21]**: IMRT: 7-field coplanar**	V95 97%	V95 95%	V95 96%
**Chera et al** [22]**: 5 equally spaced beams IMRT**		MD 31.9 Gy; V30 96.5%; Dmax 34.2 Gy; Dmin 28.1	MD 31.7 Gy; V30 97.5%; Dmax 32.7 Gy; Dmin 29.1
**Cella et al** [23]**: Forward planned IMRT**	V95 95.9%, Dmax 118%, Dmin 77%, DM 105%, IC 0.40		V95 96.8%, Dmax 111%, Dmin 79%, DM 101%, CI 0.31
**Koeck et al** [26]**: IF vs IN. Coplanar IMRT**		DM 29.7–29.8 Gy; V95% 85.3–90.5; V107% 0.4–0.5; CI 2.77–2.8, HI 0.82–0.85	DM 28.8–29.8 Gy; V95% 86.4–87.7, V107% 2.7–2.9; CI 1.24–1.25; HI 0.77–0.78
**De Sanctis et al** [28]: **5 equally spaced beams IMRT**		MD 101.4%; Dmax 108.8%; Dmin 41.4%	MD 100%: Dmax 106.6 Gy; Dmin 71.4 Gy
**Fiandra et al** [29]**. IMRT: VMAT vs Tomodirect vs Helical Tomotherapy**		MD 30.6 Gy; V95 94.8%; V107 5%; HI 0.3	MD 29.9–30.4 Gy; V95 95.4–97.4%; V107 0–5.5%; HI 0.07–0.2
**Chen et al** [30]**: IMRT Coplanar vs Non-coplanar**	D98% 34.4 Gy, MD 39.1 Gy; CI 0.47; HI 1.19		D98% 34.2–34.1 Gy, MD 38.4–38.3 Gy; CI 0.72–0.71; HI 1.13
**Antoni et al** [32]**: Helical Tomotherapy**		CI 2.4; HI 1.1; V95 93.7%	CI 1.2; HI 1.1; V95 95.8%
**Voong et al** [33]: **butterfly IMRT, multiple arcs**		CI 1.66, HI 1.14, PTV dose 32.02 Gy, V107 801.5 cm^3^	CI 1.10, HI 1.15, PTV dose 31.39 Gy
**Kriz et al** [35]: **5 beams vs 7 equally spaced beams.**	D98 92–93.5%; HI 0.14–0.47; CI 2.61–2.84		D98 92.6–93.9%; HI 0.11–0.16; CI 0.99–1.24
**Besson et al** [36]**: Tomotherapy**	MD 30.1 Gy, CI 2.9, HI 2.9; V90 98.1%, V95 94.7%, V100 63%, V107 1.2%.		MD 30.3 Gy, CI 1.4, HI 0.5;V90 99.9%, V95 98.7%, V100 52.2%, V107 0%
**Higby et al** [38]**: VMAT**	V20 35.4%, V5 52.6%, MD 13 Gy		V20 20.8%, V5 67.8%, MD 11.9 Gy

*Abbreviations*: *V* volume, *D* dose, *MD* mean dose, *HI* homogeneity index, *CI* conformity index

## Estimated late effects

As indicated by Maraldo and Specht [4], the application of late complications risk modeling into the planning process appears of high interest. This can be done through normal tissue complication probability (NTCP) models, which are mathematic models that seek to describe a dose-response relationship (i.e., the probability of a particular endpoint occurring as a function of radiation dose).

Many research groups attempted to model 3D-CRT vs. IMRT (and vs. proton therapy) effects on heart, secondary cancer, thyroid, and vessels. The NTCP of pneumonitis, pericarditis, myelitis/spinal cord necrosis, thyroiditis, esophageal stricture/perforation, and breast tissue fibrosis was investigated by Cella et al. [39] and De Sanctis et al. [28] and fractional lung damage by Goodman et al. [19]. However, these clinical endpoints are not the most frequently observed in HL survivors, and are not the most relevant concerning morbidity and mortality. Individualized estimates for the risk of development of secondary cancers and cardiovascular disease have been calculated by Koh et al. [40] Hodgson et al. [41], Weber et al. [24], Maraldo et al. [7] and Filippi et al. [8]. The potential for risk reduction with more conformal therapy was investigated by Weber et al. [42] Filippi et al. [8], Levis et al. [43] and Maraldo et al. [34]. Across these studies, IMRT techniques were not associated with an increased risk of radiation-induced secondary cancers, except for Maraldo et al. [7], where full-arc VMAT without low-dose constraints for breasts and lungs was tested. For cardiovascular disease, again, Maraldo et al. compared 3D-CRT with VMAT and found that risk estimates for cardiovascular disease were not significantly reduced with VMAT compared with 3D-CRT [7, 44]. Filippi et al. [8] and Levis et al. [43] subsequently showed that optimized multi-arcs VMAT, including low-dose constraints, was able to reduce heart disease risk in comparison with 3D-CRT, independently of the anatomical presentation (mediastinal disease alone or plus neck or unilateral axilla).

## Proposed dose objectives for organs at risk

An integral part of calculating conformal treatment plans, particularly for inverse planning IMRT, is the use of dose constraints/dose objectives for different healthy tissues. However, the dose constraints used for treatment planning of solid tumors are, in most cases, not well suited for RT planning for lymphomas, because the prescribed dose to the target is much lower than the tolerance dose for most OAR. Therefore, we preferred the term “dose objectives” as they refer to what it is the desired dose distribution to be achieved through the use of inverse planning. In general, radiation doses to all OAR should be kept as low as possible to minimize the risk of long-term complications, but some structures are more critical than others. Ideally, NTCP models for all relevant risk organs with a particular focus on the low-dose region of 20 to 40 Gy should be used for comparing each treatment plan. At present, no validated guidelines exist that allow optimization based on weighted estimates of risks of different long-term complications. We here propose a list of suggested dose objectives for critical organs at risk (heart, breasts, lungs, thyroid) to be used for IMRT optimization. Table 3 summarizes the expert panel indications for dose objectives.

**Table 3:** Suggested dose objectives for organs at risk

Structures	Recommended	Required	Avoid maximum dose in
**Heart**			
**Whole organ**	Mean < 5 Gy	Mean, 5–15 Gy	Coronary vessels
**Left ventricle**	Mean < 2 Gy	Mean, 5–10 Gy	Coronary vessels
**Breasts (whole breasts)**	Mean dose < 4 Gy	V4 < 50%	Glandular tissue
**Lungs (minus PTV)**	V5 < 55%	V_5_ 55–60%	
	V20 < 30%	V20 < 35%	
	Mean dose < 10 Gy	Mean < 13.5 Gy	
**Thyroid**	V5 < 93% V20 < 82% V25 < 63% V30 < 62% 2.2 ml < 25 Gy	V25 < 70%	Whole thyroid

### Heart

The association between RT and cardiac complications is now well known for patients treated for mediastinal lymphomas. However, cardiac complications due to chest irradiation have been considered rare and insignificant [45] for quite a long time, though the first report on potential adverse effects of cardiac irradiation was published in 1897 [46]. The first detailed and reliable description of radio-induced heart disease (RIHD) dates back to occasional clinical observations published 40 to 50 years ago [47, 48]. Two subsequent publications by Hancock et al. from Stanford University, in a large cohort of adult and pediatric patients cured for HL, established that the risk of heart disease is related to the dose received [49, 50]. Since then, several studies confirmed the existence of a causal link between thoracic irradiation and heart toxicity in long-term survivors treated at a young age for lymphoproliferative disorders or pediatric neoplasms [51, 52], and for breast cancer [53]. Acute effects (within 6 months of completion of treatment) are mainly represented by pericarditis, which is usually transient and easily treatable with anti-inflammatory therapy. Late complications are entirely different events, such as chronic heart failure, unstable angina, myocardial infarction, valve failure, and arrhythmia; late effects occur many years after RT completion, usually after 20–30 years [54, 55]. Based on these historical reports, the long-term risk of coronary artery disease and chronic heart failure for HL patients receiving combined chemo-radiotherapy is increased 5 and 7 times, respectively, compared to healthy people. The occurrence of late radiation-induced heart disease led to an intense debate about the risk-benefit balance of the use of RT for mediastinal lymphomas. A few randomized trials have attempted to omit RT from the first-line therapy, intending to reduce life-threatening long-term complications (mainly heart events and second neoplasms), while accepting a slight (albeit statistically significant) reduction in disease control [56–59]. However, the constant improvement of RT techniques, the reduction of treatment volumes through the introduction of the ISRT and INRT concepts, and the significant decrease in prescription doses, from 40 to 44 Gy to the current 20–30 Gy dose range, have partially modified the clinical risk of RIHD [60]. In particular, the use of IMRT and daily image-guidance with IGRT techniques, as well as the integration of respiratory gating, emerged as factors that could drastically reduce mean and maximum heart dose. When a modern planning and dose delivery is adopted, it seems reasonable to keep the mean heart dose (MHD) below 5 Gy, as suggested by Maraldo and Ng [60].

The recent advances in the identification of the heart substructures, guided by dedicated atlases [61, 62] and in the accurate estimation of their displacement related to the heart motion [63, 64], suggest performing detailed contouring of valves, chambers and coronary arteries. Given the irregular and variable shape of HL PTV, which is unique for every single patient in relation to the variable extension of mediastinal involvement and its proximity to the heart, the adoption of more conformal techniques allows a steep dose gradient. It may lead to a more heterogeneous dose distribution across heart substructures. As a result, the relationship between MHD and mean substructures dose is weak, and the adoption of MHD alone is becoming inadequate, if not meaningless, in the modern radiotherapy era [65].

A recent contribution from Princess Margaret Hospital Cancer Center in Toronto has firstly shown that a risk model for ischemic disease, including coronary artery variables, is superior to a model purely based on the mean heart dose [66]. In that report, V_5_ of left anterior descending and V_20Gy_ of left circumflex were the best predictors of coronary artery disease. Unfortunately, no specific constraints were provided by the authors.

The ongoing CARDIOCARE project (Clinical Trial identifier: NCT03480087) has firstly shown a significant impairment of left ventricular systolic function, evaluated with global longitudinal strain, in patients receiving an MHD > 5 Gy and a mean left ventricular dose > 2 Gy [67], thus suggesting an indicative dose limit for ventricular dysfunction.

There is no best IMRT solution to spare the heart and its substructures. Many reports have compared different approaches (HT, static IMRT, multiple arcs VMAT), all concluding that the plans should be tailored to every single patient and his/her clinical features. In general, a multi-arc solution appears more effective in reducing the doses to the heart substructures, in particular to the coronary arteries, compared to static IMRT or HT, with a slight increase of the low-dose component for the breasts and the lungs [29, 33]. Therefore, comparative planning between different IMRT solutions is strongly advised in order to guide the clinical judgment and calibrate the treatment to patients’ needs, adopting a personalized approach.

The following are practical indications on contouring, radiation technique, and dose objectives derived from different studies and expert consensus:

- We recommend the contouring of the heart as a whole and all cardiac sub-structures (coronary arteries, valves, and cardiac chambers), as indicated in the atlas published by Feng et al. [61] from Michigan University. An expansion margin for coronary arteries could be considered, as published by Levis et al. (left main trunk: 3 mm; left anterior descending: 5 mm; circumflex: 4 mm; right coronary artery: 5 mm) [63].
- The contouring of all cardiac structures allows customizing the radiotherapy treatment plan and optimizing the saving of areas considered most relevant from the clinical point of view. In this regard, the saving of coronary arteries and left ventricle are considered a priority for their clinical relevance (risk of coronary artery disease and chronic heart failure in the medium to long term).
- The risk of radio-induced cardiotoxicity is higher for patients aged > 35 years, for whom maximum attention must be paid to saving the heart and its structures.
- There is an Excess Relative Risk (ERR) per Gy of 7.4% in ischemic heart disease risk [9] for the dose received by the heart and an ERR per Gy of 9% in heart failure for the dose received by the left ventricle [68]. These data indicate a clear dose-response effect, and suggest that doses should be kept as low as reasonable to every cardiac substructure. For valvular dysfunction, the dose-response relationship is exponential, increasing steeply above 20 Gy [11]
- Multi-arcs VMAT solutions seem to provide the best dosimetric benefit to the heart and its substructures. Comparative planning between different IMRT solutions is strongly recommended in every single case to tailor the treatment to patients’ needs.

### Breasts

Longitudinal epidemiological studies on large patients’ cohorts treated with chemotherapy and radiation therapy for early-stage Hodgkin’s lymphoma revealed the carcinogenic potential of fractionated mediastinal RT, recording a substantial increase in the risk of secondary breast and lung cancers, especially for young women (under 30 years old) [5, 69–72]. The risk is maximum with the use of broad fields (mantle) and high-dose. Exposure of the breast tissue to high-intermediate dose, especially on the inner quadrants, is associated with cellular damage that is not easily repairable and, with a multifactorial etiology, may lead to developing a radiation-induced breast tumor.

While a reduction in volumes is associated with an apparent reduction in carcinogenic damage (from mantle fields to involved fields), the exact dose distribution to mammary glands plays an important role. Progressive dose reduction from 44 to 36 Gy, and then from 30 to 20 Gy is also associated with substantial risk reduction, as suggested by various biological models and confirmed by early clinical data. The irradiation technique can have an impact as dose distribution is modified, reducing the proportion of parenchyma receiving therapeutic doses (30 Gy, as was the case with the AP-PA approach) but increasing the proportion receiving doses below 5 Gy. The risk of carcinogenesis at fractionated low-dose radiation is difficult to model; however, most studies converge on a linear relationship, with apparent reduction at low-dose given the partial exposure [5, 6, 73–75].

With the use of various IMRT techniques, breasts DVHs change shape, increasing the low-dose component and reducing the high-dose component, even with the same average dose. Here, we propose the adoption of specific dose objectives for the mammary glands, based on the low-dose limit of 4 Gy (below which the relative risk of carcinogenesis, in pediatric cohorts and therefore at higher intrinsic risk, is about 1.3) [76]. By applying this low-dose constraint, the risk associated with IMRT appears similar to what expected with more classical 3D approaches: this is possible because the linear model assigns a proportionally lower carcinogenic risk to low-dose than to high-dose. In other words, it is acceptable to expose larger volumes of the breast to doses below 4 Gy when reducing the proportion of tissue receiving doses of 20–30 Gy. The main advantage is to achieve a better heart sparing without increasing carcinogenic risk. The following are some practical indications:

- We recommend the contouring of both breasts as a single OAR
- Patients under 20 years old are at the higher risk; the risk is still elevated compared with baseline for patients aged 20–30 years [73]. For patients above 30 years old, the risk is lower and cardiac sparing may take precedence.
- It is preferable a technical approach that avoids a direct entrance through the mammary glands, preferring anteroposterior non-coplanar arches plus a small anterior arc (also to the benefit of the lungs bilaterally), or single arc but with constraints on the lung and breasts such as to avoid low-dose spread bilaterally.
- It is recommended to compare rival plans, in light of the desired balance between the risk of cardiac, pulmonary, and breast toxicity. This customization is essential, given the tremendous anatomical variability of the target volumes in HL.

### Lungs

The correlation between the occurrence and grade of lung toxicity and irradiated lung volumes in lymphomas has been initially assessed in patients treated with IFRT in the dose range 30–45 Gy [77]. In these studies, the dosimetric parameters that emerged as mostly related to the risk of pneumonia were mean lung dose (MLD), V20, and V30, similarly to what found for lung cancer. Most of the patients were treated with anteroposterior techniques, exposing to full dose areas around the mediastinum and the apexes. In all these studies, the risk of toxicity was significantly increased in the subgroup of refractory/relapsed patients. Pinnix et al. analyzed 150 pts. with mediastinal lymphomas treated with ISRT/IMRT [78]. Symptomatic pneumonia was found in 14% of patients, with 6.7% G3, after a median follow-up of 2.04 months (range 0.33–9.18). The incidence was 10% in patients treated with first-line RT and 25% in patients in whom RT was performed at relapse/salvage. V20 > 30%, as well as MLD > 13.5 Gy and V5 > 55%, were all associated with a higher risk of pneumonia. Other factors such as age, race, disease stage, gender, type of chemotherapy, previous bleomycin toxicity, bulky disease, tobacco habit, previous asthmatic disease, or chronic obstructive pulmonary disease, were not significant. We recommend to delineate both lungs and consider both as a single OAR, subtracting the planning target volume (PTV).

### Thyroid

Hypothyroidism was a quite common late effect after the irradiation of the upper chest and neck areas at doses of above 30 Gy, with a cumulative risk of 40–60% [79–81]. A study including patients treated with two opposing AP-PA fields [82] showed that the best predictor for hypothyroidism was V30, with a 62.5% cut-off. In detail, the risk of hypothyroidism was 11.5%, with a V30 < 62.5 and 70.8% with a V30 above this threshold. The same study also identified a threshold value for V20: in patients with a V20 higher or lower than 82.4%, the risk of hypothyroidism was 60.7 and 13.6%, respectively. A more recent study [83] assessed the risk of hypothyroidism in patients treated with IMRT: for V25, the threshold value was 63.5% (37% vs. 80% hypothyroidism), while the cut-off for V30 was 62%. Another parameter found to be related to the risk of functional damage was the amount of thyroid tissue receiving less than 25Gy, with a threshold value at 2.2 ml. Patients with a smaller thyroid gland appeared to be at higher risk, with a cut-off value of 11.2 ml.

#### Motion management

Radiotherapy evolutions for mediastinal lymphomas have seen the progressive spread of breathing control methods, or so-called “breath-holding” techniques. This approach encompasses two techniques: Active Breathing Control (ABC) and Deep Inspiration Breath-Hold (DIBH). The ABC system automatically synchronizes breathing with the treatment unit, being able to hit the target in a specific phase of the respiratory cycle, where OAR are located in a more favorable position. The DIBH system requires the patient to maintain the same level of forced inhalation during the simulation and treatment phases, intending to ensure RT delivery only in a pre-defined and reproducible situation of OAR displacement (heart, breasts). With both techniques, the respiratory motion range may be reduced to less than 5 mm (rather than 10–15 mm).

The use of DIBH and image-guidance for mediastinal lymphomas is limited but well supported by high-quality studies [34, 84, 85]. All reports have shown that the DIBH technique can reduce the dose to heart, breasts, and lungs, and this effect is mainly due to the displacement of OAR in the deep inhalation phase secondary to the forced increase of the lung volume. The benefit achievable with DIBH seems independent by the technique used [34]. A few Centers apply smaller margins for PTV in the context of DIBH, further accentuating its benefit for the heart and lungs.

The use of 4D-CT allows for an individualization of PTV margins, according to the principle of “internal target volume” (ITV); even if mediastinal structures motion is limited in comparison with lungs, this method may be helpful.

#### Use of IGRT

Image-guided radiotherapy (IGRT) is a process involving the use of imaging during RT delivery, intending to improve the accuracy of treatment. IGRT allows for a real-time or a near real-time visualization of anatomical details, as well as changes in tumor volume and position. In general, IGRT uses different imaging modalities to locate the target before and during each treatment session. IGRT has gained popularity and rapid spread over the last decade, especially in some clinical scenarios such as lung, prostate, head and neck, and gastrointestinal tract cancers; however, the role of IGRT in the management of lymphomas is not well defined. A prospective trial tested the use of IGRT for HL, showing the benefit of combining DIBH with IGRT [86].

The association between IGRT and IMRT seems rational and mandatory, especially Cone-beam CT (CBCT) or MV-CT, as it allows for margin reduction, and ideally could be combined with either 4D-CT individualized margins or respiratory gating. A volumetric scan with kV Cone-beam delivers on average an integral dose of about 1–10 cGy per acquisition [87–89]. Recent radiobiological models generally indicate a negligible impact of these fractional dose levels on the risk of second neoplasms.

Being aware that the most appropriate modalities for IGRT are a prerogative of each Centre, as they strictly depend on the CTV-PTV margins used and the immobilization systems, extended use of IGRT is recommended when using IMRT in combination with ISRT-INRT for mediastinal lymphomas.

## Discussion and conclusions

Hodgkin’s lymphoma clinical and anatomical presentations are widely variable, as are treatment volumes and RT planning and delivery techniques. The expert panel accounted for the difficulties in the correct interpretation of independent studies applying different target volume definitions, OAR definition and contouring, and optimization priorities, which were often not defined. This variability highlights the variation in OAR dose based on the priority given to each structure during planning, which varies on a case-by-case basis, dependent on the individual’s health status, age, sex, and life expectancy. Also, there is an inconsistent reporting of dosimetric and plan evaluation parameters, and the results of a single study may apply to only a limited sample of patients (e.g., favorable, females, young, mediastinal mass).

However, we observed that: a) IMRT provides a substantially lower dose to the healthy tissues compared with AP-PA or multi-fields 3D-CRT; however, the individual variation is considerable, and some patients will still receive a high-dose because of the proximity of the target volume to the OAR(s) investigated; b) IMRT should be seen as one treatment modality of several possible, each with different dose distributions; c) the choice should be made individually, because of a lack of superiority of one technique over the others when multiple OAR are considered simultaneously; d) in the majority of the reported experiences, CTV-PTV margins were smaller than in the past, according to the systematic use of IGRT, with a significant impact on treated volumes and, consequently, on the ability of IMRT to spare OAR, and e) organ motion management techniques have been adopted by a few Centers but showed their potential in improving dose distribution, further reducing heart and breast dose.

In the near future, proton therapy (PT) will be an option, especially for younger patients. The use of PT for lymphomas engaging the mediastinum is promising, and treatment techniques continue to evolve. However, the limited availability of PT calls for case selection based on a clear understanding of which cases will derive most benefit from protons therapy as compared to advanced photon techniques, as underlined by Dabaja et al. [90]. Moreover, the combination of photons with protons, the use of gating or breath-hold, the use of IGRT with IMPT (intensity-modulated PT) might open a new window of opportunity, particularly for cardiac substructures sparing.

**Fig. 1:**
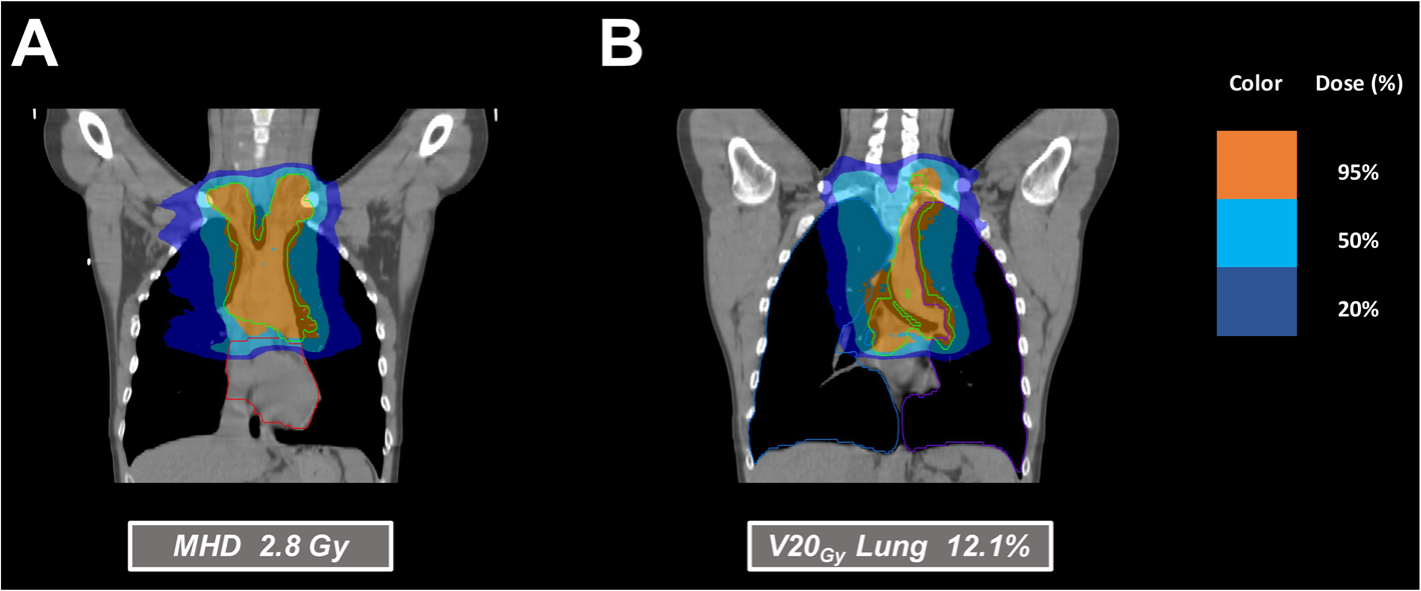
A multi-arcs VMAT plan in a male patient, in DIBH, where heart sparing has the highest priority: heart displacement combined with optimized VMAT achieves low mean heart dose (MHD) (**a**) combined with low V20_Gy_ (**b**)

**Fig. 2:**
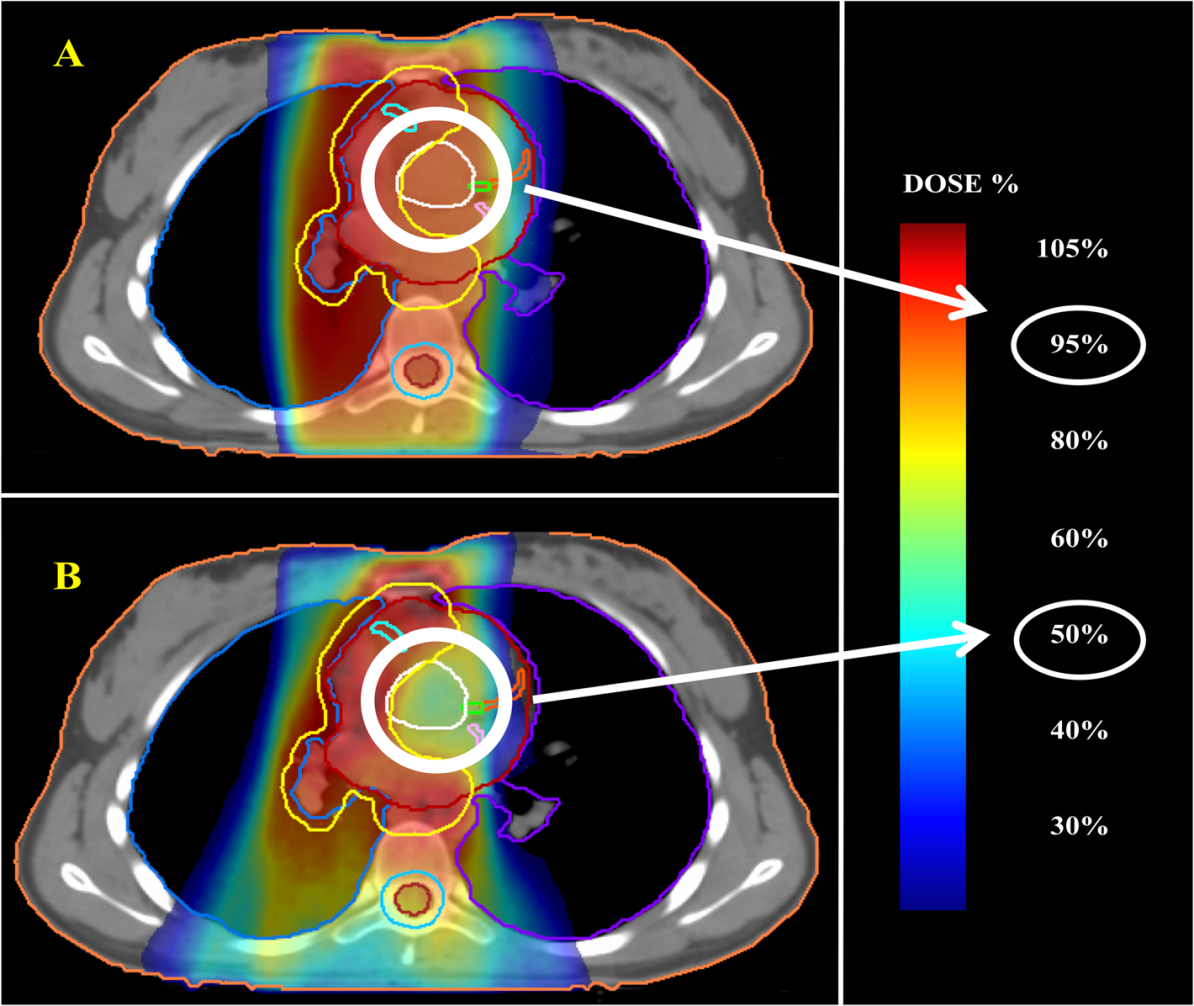
Comparative planning between AP-PA (**a**) and butterfly VMAT (**b**) in a male patient, showing the achievable dose distribution on single heart substructures (the aortic valve in white, the left main trunk in green, the left anterior descending artery in orange and the circumflex artery in pink)

**Fig. 3:**
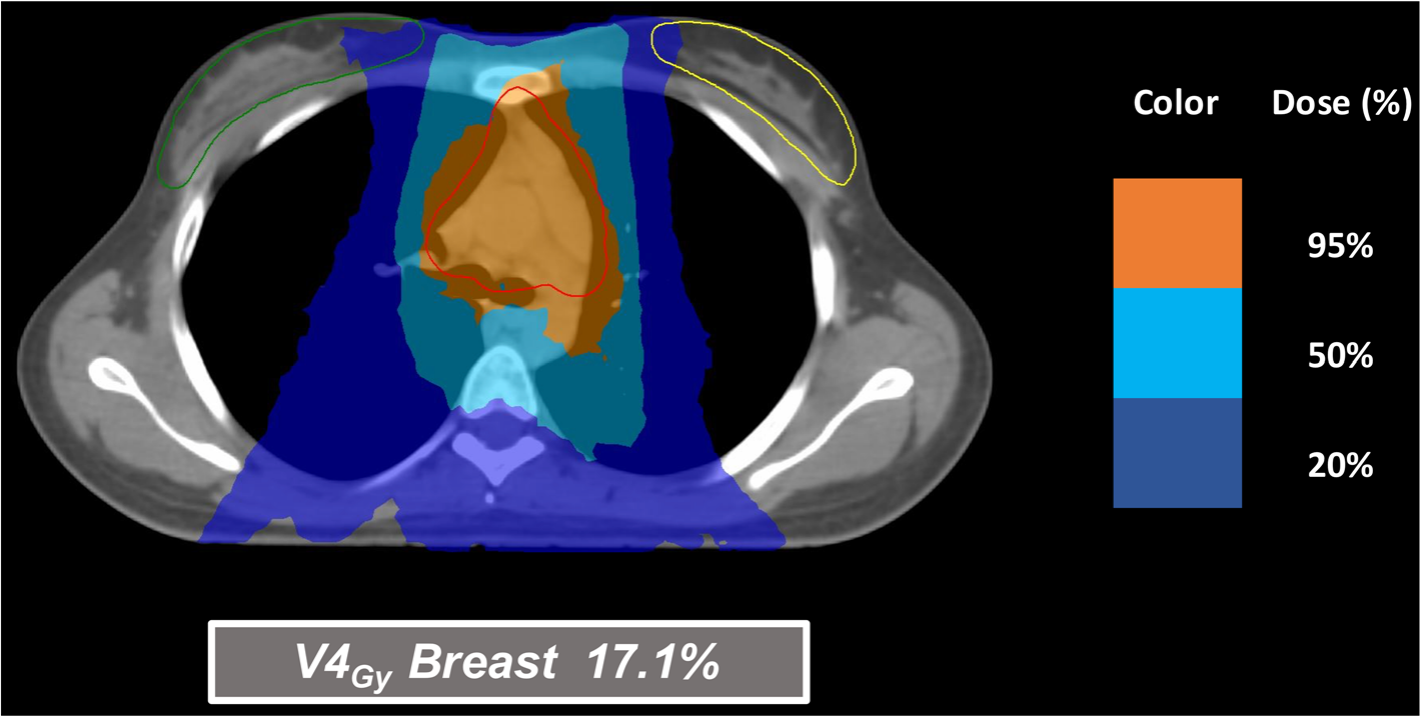
A case of a young female patient, where breast-sparing has the highest priority, showing in axial view an optimized multi-arcs VMAT plan with good PTV coverage, low breasts V4_Gy_ and acceptable heart and lungs dose

Figures 1, 2 and 3 illustrate a few examples of the achievable dose distributions with multi-arcs VMAT on whole heart, heart substructures, lungs and breasts in different clinical cases (male and female patients).

In conclusion, the FIL expert panel recommends the use of IMRT for mediastinal lymphomas, with various technical solutions, incorporating specific dose objectives into the planning process, and considering individual risks. The panel also recommends the use of daily IGRT and, when feasible, support the implementation of breath-control techniques.

## Data Availability

Not applicable.

## Abbreviations

3D-CRT: Three-dimension conformal radiotherapy
4D-CT: Four-dimensions computed tomography
ABC: Active breathing coordinator
AP-PA: Antero-posterior/postero-anterior
COPD: Chronic obstructive pulmonary diseae
CT: Computed tomography
CT-PET: Computed tomography_positron emission tomography
DIBH: Deep-inspiration breath hold
EORTC: European organization for research and treatment of cancer
FIL: Fondazione Italiana Linfomi
HL: Hodgkin’s lymphoma
HT: Helical tomotherapy
IGRT: Image-guided radiotherapy
ILROG: International lymphoma radiation oncology group
IMPT: Intensity-modulated proton therapy
IMRT: Intensity-modulated radiotherapy
INRT: Involved-node radiotherapy
ISRT: Involved-site radiation therapy
ITV: Internal target volume
MHD: Mean heart dose
MLD: Mean lung dose
NTCP: Normal tissue complication probability
OAR: Organs-at-risk
PT: Proton therapy
PTV: Planning target volume
RIHD: Radiation-induced heart disease
VMAT: Volumetric modulated arc therapy

## References

[1] Eichenauer DA, Aleman BMP, André M, Federico M, Hutchings M, Illidge T, ESMO Guidelines Working Group (2018). Hodgkin lymphoma: ESMO clinical practice guidelines for diagnosis, treatment, and follow-up. Ann Oncol.

[2] Filippi AR, Levis M, Parikh R, Hoppe B (2017). Optimal therapy for early-stage Hodgkin's lymphoma: risk adapting, response adapting, and role of radiotherapy. Curr Oncol Rep.

[3] Hall EJ, Wuu CS (2003). Radiation-induced second cancers: the impact of 3D-CRT and IMRT. Int J Radiat Oncol Biol Phys.

[4] Maraldo MV, Specht L (2014). A decade of comparative dose planning studies for early-stage Hodgkin lymphoma: what can we learn?. Int J Radiat Oncol Biol Phys.

[5] Berrington de Gonzalez A, Gilbert E, Curtis R (2013). Second solid cancers after radiation therapy: a systematic review of the epidemiologic studies of the radiation dose-response relationship. Int J Radiat Oncol Biol Phys.

[6] Filippi AR, Vanoni V, Meduri B (2018). Intensity modulated radiation therapy and second cancer risk in adults. Int J Radiat Oncol Biol Phys.

[7] Maraldo MV, Brodin NP, Aznar MC (2013). Estimated risk of cardiovascular disease and second cancers with modern highly conformal radiotherapy for early stage mediastinal Hodgkin lymphoma. Ann Oncol.

[8] Filippi AR, Ragona R, Piva C (2015). Optimized volumetric modulated arc therapy versus 3D-CRT for early stage mediastinal Hodgkin lymphoma without axillary involvement: a comparison of second cancers and heart disease risk. Int J Radiat Oncol Biol Phys.

[9] van Nimwegen FA, Schaapveld M, Cutter DJ (2016). Radiation dose-response relationship for risk of coronary heart disease in survivors of Hodgkin lymphoma. J Clin Oncol.

[10] Moignier A, Broggio D, Derreumaux S (2015). Coronary stenosis risk analysis following Hodgkin lymphoma radiotherapy: a study based on patient specific artery segments dose calculation. Radiother Oncol.

[11] Cutter DJ, Schaapveld M, Darby SC (2015). Risk for valvular heart disease after treatment for Hodgkin lymphoma. J Natl Cancer Inst.

[12] Hoppe R, Advani RH, Ai WZ (2018). NCCN guidelines insights: Hodgkin lymphoma, version 1.2018. J Natl Compr Can Net.

[13] Girinsky T, van der Maazen R, Specht L (2006). Involved-node radiotherapy (INRT) in patients with early Hodgkin lymphoma: concepts and guidelines. Radiother Oncol.

[14] Specht L, Yahalom J, Illidge T (2014). Modern radiation therapy for Hodgkin lymphoma: field and dose guidelines from the International Lymphoma Radiation Oncology Group (ILROG). Int J Radiat Oncol Biol Phys.

[15] Filippi AR, Ciammella P, Piva C (2014). Involved-site image-guided intensity-modulated radiotherapy vs. 3D conformal radiotherapy in supra-diaphragmatic early-stage Hodgkin's lymphoma. Int J Radiat Oncol Biol Phys.

[16] Campbell BA, Voss N, Pickles T (2008). Involved-nodal radiation therapy as a component of combination therapy for limited-stage Hodgkin’s lymphoma: a question of field size. J Clin Oncol.

[17] Paumier A, Ghalibafian M, Beaudre A (2011). Involved-node radio- therapy and modern radiation treatment techniques in patients with Hodgkin lymphoma. Int J Radiat Oncol Biol Phys.

[18] Maraldo MV, Aznar MC, Vogelius IR (2013). Involved node radiation therapy: an effective alternative in early-stage Hodgkin's lymphoma. Int J Radiat Oncol Biol Phys.

[19] Goodman KA, Toner S, Hunt M, Wu EJ, Yahalom J (2005). Intensity-modulated radiotherapy fol lymphoma involving the mediastinum. Int J Radiat Oncol Biol Phys.

[20] Girinsky T, Pichenot C, Beaudre A, Ghalibafian M, Lefkopoulos D (2006). Is intensity-modulated radiotherapy better than conventional radiation treatment and three-dimensional conformal radiotherapy for mediastinal masses in patients with Hodgkin's disease, and is there a role for beam orientation optimization and dose constraints assigned to virtual volumes?. Int J Radiat Oncol Biol Phys.

[21] Nieder C, Schill S, Kneschaurek P, Molls M (2007). Comparison of three different mediastinal radiotherapy techniques in female patients: impact on heart sparing and dose to the breasts. Radiother Oncol.

[22] Chera BS, Rodriguez C, Morris CG, Louis D, Yeung D, Li Z, Mendenhall NP (2009). Dosimetric comparison of three different involved nodal irradiation techniques for stage II Hodgkin's lymphoma patients: conventional radiotherapy, intensity-modulated radiotherapy, and three-dimensional proton radiotherapy. Int J Radiat Oncol Biol Phys.

[23] Cella L, Liuzzi R, Magliulo M, Conson M, Camera L, Salvatore M, Pacelli R (2010). Radiotherapy of large target volumes in Hodgkin's lymphoma: normal tissue sparing capability of forward IMRT versus conventional techniques. Radiat Oncol.

[24] Weber DC, Johanson S, Peguret N (2011). Predicted risk of radiation-induced cancers after involved field and involved node radiotherapy with or without intensity modulation for early-stage hodgkin lymphoma in female patients. Int J Radiat Oncol Biol Phys.

[25] Hoppe BS, Flampouri S, Su Z, Latif N, Dang NH, Lynch J, Joyce M, Sandler E, Li Z, Mendenhall NP (2012). Effective dose reduction to cardiac structures using protons compared with 3DCRT and IMRT in mediastinal Hodgkin lymphoma. Int J Radiat Oncol Biol Phys.

[26] Koeck J, Abo-Madyan Y, Lohr F, Stieler F, Kriz J, Wenz F, Eich HT (2012). Radiotherapy for early mediastinal Hodgkin lymphoma according to the German Hodgkin study group (GHSG): the roles of intensity-modulated radiotherapy and involved-node radiotherapy. Int J Radiat Oncol Biol Phys.

[27] Campbell BA, Hornby C, Cunninghame J (2012). Minimising critical organ irradiation in limited stage Hodgkin lymphoma: a dosimetric study of the benefit of involved node radiotherapy. Ann Oncol.

[28] De Sanctis V, Bolzan C, D'Arienzo M (2012). Intensity modulated radiotherapy in early stage Hodgkin lymphoma patients: is it better than three dimensional conformal radiotherapy?. Radiat Oncol.

[29] Fiandra C, Filippi AR, Catuzzo P (2012). Different IMRT solutions vs. 3D-conformal radiotherapy in early stage Hodgkin's lymphoma: dosimetric comparison and clinical considerations. Radiat Oncol.

[30] Chen X, Jin D, Wang S, Li M, Huang P, Dai J (2012). Noncoplanar intensity-modulated radiation therapy for young female patients with mediastinal lymphoma. J Appl Clin Med Phys.

[31] Cella L, Conson M, Pressello MC (2013). Hodgkin's lymphoma emerging radiation treatment techniques: trade-offs between late radio-induced toxicities and secondary malignant neoplasms. Radiat Oncol.

[32] Antoni D, Natarajan-Ame S, Meyer P (2013). Contribution of three-dimensional conformal intensity-modulated radiation therapy for women affected by bulky stage II supradiaphragmatic Hodgkin disease. Radiat Oncol.

[33] Voong KR, McSpadden K, Pinnix CC, Shihadeh F, Reed V, Aristophanous M, Dabaja BS (2014). Dosimetric advantages of a "butterfly" technique for intensity-modulated radiation therapy for young female patients with mediastinal Hodgkin's lymphoma. Radiat Oncol.

[34] Aznar MC, Maraldo MV, Schut DA (2015). Minimizing late effects for patients with Mediastinal Hodgkin lymphoma: deep inspiration breath-hold, IMRT, or both?. Int J Radiat Oncol Biol Phys.

[35] Kriz J, Spickermann M, Lehrich P (2015). Breath-hold technique in conventional APPA or intensity-modulated radiotherapy for Hodgkin's lymphoma: comparison of ILROG IS-RT and the GHSG IF-RT. Strahlenther Onkol.

[36] Besson N, Pernin V, Zefkili S, Kirova YM (2016). Evolution of radiation techniques in the treatment of mediastinal lymphoma: from 3D conformal radiotherapy (3DCRT) to intensity-modulated RT (IMRT) using helical tomotherapy (HT): a single-Centre experience and review of the literature. Br J Radiol.

[37] Horn S, Fournier-Bidoz N, Pernin V (2016). Comparison of passive-beam proton therapy, helical tomotherapy and 3D conformal radiation therapy in Hodgkin’s lymphoma female patients receiving involved-field or involved site radiation therapy. Cancer Radiother.

[38] Higby C, Khafaga Y, Al-Shabanah M, Mousa A, Ilyas M, Nazer G, Khalil EM (2016). Volumetric-modulated arc therapy (VMAT) versus 3D-conformal radiation therapy in supra-diaphragmatic Hodgkin's lymphoma with mediastinal involvement: a dosimetric comparison. J Egypt Natl Canc Inst.

[39] Cella L, Liuzzi R, D’Avino V (2014). Pulmonary damage in Hodgkin’s lymphoma patients treated with sequential chemo-radiotherapy: predictors of radiation-induced lung injury. Acta Oncol.

[40] Koh ES, Sun A, Tran TH (2006). Clinical dose-volume histogram analysis in predicting radiation pneumonitis in Hodgkin’s lymphoma. Int J Rad Oncol Biol Phys.

[41] Hodgson DC, Koh ES, Tran TH (2007). Individualized estimates of second cancer risks after contemporary radiation therapy for Hodgkin lymphoma. Cancer.

[42] Weber DC, Peguret N, Dipasquale G, Cozzi L (2009). Involved-node and involved-field volumetric modulated arc vs. fixed beam intensity-modulated radiotherapy for female patients with early-stage supra-diaphragmatic Hodgkin lymphoma: a comparative planning study. Int J Radiat Oncol Biol Phys.

[43] Levis M, Filippi AR, Fiandra C (2019). Inclusion of heart substructures in the optimization process of volumetric modulated arc therapy techniques may reduce the risk of heart disease in Hodgkin's lymphoma patients. Radiother Oncol.

[44] Maraldo MV, Brodin NP, Vogelius IR (2012). Risk of developing cardiovascular disease after involved-node radiotherapy versus mantle field for Hodgkin lymphoma. Int J Radiat Oncol Biol Phys.

[45] Leach JEL (1943). Effect of roentgen therapy on the heart: clinical study. Arch Intern Med.

[46] Seguy G, Quenisset F (1897). Action des rayons X sur le Coeur. Comptes rendus hebdomadaires des séances de l’Académie des sciences.

[47] Dollinger MR, Lavine DM, Foye LV (1966). Myocardial infarction due to post-irradiation fibrosis of the coronary arteries. Case of successfully treated Hodgkin’s disease with lower esophageal involvement. JAMA.

[48] Yahalom J, Hasin Y, Fuks Z (1983). Acute myocardial infarction with normal coronary arteriogram after mantle field radiation therapy for Hodgkin’s disease. Cancer.

[49] Hancock SL, Tucker MA, Hoppe RT (1993). Factors affecting late mortality from heart disease after treatment of Hodgkin’s disease. JAMA.

[50] Hancock SL, Donaldson SS, Hoppe RT (1993). Cardiac disease following treatment of Hodgkin’s disease in children and adolescents. J Clin Oncol.

[51] Mulrooney DA, Yeazel MW, Kawashima T (2009). Cardiac outcomes in a cohort of adult survivors of childhood and adolescent cancer: retrospective analysis of the childhood Cancer survivor study cohort. BMJ.

[52] Tukenova M, Guibout C, Oberlin O (2010). Role of cancer treatment in long term overall and cardiovascular mortality after childhood cancer. J Clin Oncol.

[53] Darby SC, Ewertz M, McGale P (2013). Risk of ischemic heart disease in women after radiotherapy for breast cancer. N Engl J Med.

[54] Aleman BPM, van den Belt-Dusebout AW, De Bruin ML (2007). Late cardiotoxicity after treatment for Hodgkin lymphoma. Blood.

[55] Jaworski C, Mariani JA, Wheeler G (2013). Cardiac complications of thoracic irradiation. JACC.

[56] Meyer RM, Gospodarowicz MK, Connors JM (2012). ABVD alone versus radiation-based therapy in limited-stage Hodgkin’s lymphoma. N Engl J Med.

[57] Raemaekers JM, André MP, Federico M (2014). Omitting radiotherapy in early positron emission tomography-negative stage I/II Hodgkin lymphoma is associated with an increased risk of early relapse: clinical results of the preplanned interim analysis of the randomized EORTC/LYSA/FIL H10 trial. J Clin Oncol.

[58] Radford J, Illidge T, Counsell N (2015). Results of a trial of PET-directed therapy for early-stage Hodgkin’s lymphoma. N Engl J Med.

[59] Fuchs M, Goergen H, Kobe C (2019). Positron emission tomography-guided treatment in early-stage favorable Hodgkin lymphoma: final results of the international, randomized phase III HD16 trial by the German Hodgkin study group. J Clin Oncol.

[60] Maraldo MV, Ng AK (2016). Minimizing cardiac risks with contemporary radiation therapy for Hodgkin lymphoma. J Clin Oncol.

[61] Feng M, Moran JM, Koelling T (2011). Development and validation of a heart atlas to study cardiac exposure to radiation following treatment for breast cancer. Int J Radiat Oncol Biol Phys.

[62] Duane F, Aznar MC, Bartlett F (2017). A cardiac atlas for contouring. Radiother Oncol.

[63] Levis M, De Luca V, Fiandra C (2018). Plan optimization for mediastinal radiotherapy: estimation of coronary arteries motion with ECG-gated cardiac imaging and creation of compensatory expansion margins. Radiother Oncol.

[64] Kataria T, Bisht SS, Gupta D (2016). Quantification of coronary artery motion and internal risk volume from ECG gated radiotherapy planning scans. Radiother Oncol.

[65] Hoppe BS, Bates JE, Mendenhall NP, et al. The meaningless meaning of mean heart dose in mediastinal lymphoma in the modern radiotherapy era. Pract Radiat Oncol. 2019; (in press). 10.1016/j.prro.2019.09.015 31586483

[66] Hahn E, Jiang H, Ng A (2017). Late cardiac toxicity after mediastinal radiation therapy for Hodgkin lymphoma: contributions of coronary artery and whole heart dose-volume variables to risk prediction. Int J Radiat Oncol Biol Phys.

[67] Levis M, De Luca V, Bartoncini S (2018). A prospective, observational study evaluating early subclinical Cardiotoxicity with global longitudinal strain imaging in lymphoma patients treated with chemotherapy +/− Mediastinal radiotherapy: the CARDIOCARE project. Int J Radiat Oncol Biol Phys.

[68] van Nimwegen FA, Ntentas G, Darby SC (2018). Risk of heart failure in survivors of Hodgkin lymphoma: effects of cardiac exposure to radiation and anthracyclines. Blood.

[69] Van Leeuwen FE, Klokman WJ, Veer MB (2000). Long-term risk of second malignancy in survivors of Hodgkin’s disease treated during adolescence or young adulthood. J Clin Oncol.

[70] Ng AK, Bernardo MV, Weller E (2002). Second malignancy after Hodgkin’s disease treated with radiation therapy with or without chemotherapy: long term risks and risk factors. Blood.

[71] Dores GM, Metayer C, Curtis RE (2002). Second malignant neoplasms among long-term survivors of Hodgkin’s disease: a population-based evaluation over 25 years. J Clin Oncol.

[72] Travis LB, Hill D, Dores GM (2005). Cumulative absolute breast cancer risk for young women treated for Hodgkin lymphoma. J Natl Cancer Inst.

[73] Swerdlow AJ, Cooke R, Bates A (2012). Breast cancer risk after supradiaphragmatic radiotherapy for Hodgkin's lymphoma in England and Wales: a National Cohort Study. J Clin Oncol.

[74] Chargari C, Goodman K (2016). Diallo et al. risk of second cancers in the era of modern radiation therapy: does the risk benefit analysis overcome theoretical models?. Cancer Metastasis Rev.

[75] Schneider Uwe, Walsh Linda (2017). Risk of secondary cancers: Bridging epidemiology and modeling. Physica Medica.

[76] Guibout C, Adjadj E, Rubino C (2005). Malignant breast tumors after radiotherapy for a first cancer during childhood. J Clin Oncol.

[77] Fox AM, Dosoretz AP, Mauch PM (2012). Predictive factors for radiation pneumonitis in Hodgkin lymphoma patients receiving combined-modality therapy. Int J Radiat Oncol Biol Phys.

[78] Pinnix CC, Smith GL, Milgrom S (2015). Predictors of radiation pneumonitis in patients receiving intensity modulated radiation therapy for Hodgkin and non-Hodgkin lymphoma. Int J Rad Oncol Biol Phys.

[79] Sklar C, Whitton J, Mertens A (2000). Abnormalities of the thyroid in survivors of Hodgkin's disease: data from the childhood Cancer survivor study. J Clin Endocrinol Metab.

[80] Constine LS, Donaldson SS, McDougall IR, Cox RS, Link MP, Kaplan HS (1984). Thyroid dysfunction after radiotherapy in children with Hodgkin's disease. Cancer.

[81] Hancock SL, Cox RS, McDougall IR (1991). Thyroid diseases after treatment of Hodgkin's disease. N Engl J Med.

[82] Cella L, Conson M, Caterino M (2012). Thyroid V30 predicts radiation-induced hypothyroidism in patients treated with sequential chemo-radiotherapy for Hodgkin's lymphoma. Int J Radiat Oncol Biol Phys.

[83] Pinnix CC, Cella L, Andraos TY (2018). Predictors of hypothyroidism in Hodgkin lymphoma survivors after intensity modulated versus 3-dimensional radiation therapy. Int J Radiat Oncol Biol Phys.

[84] Paumier A, Ghalibafian M, Gilmore J (2012). Dosimetric benefits of intensity-modulated radiotherapy combined with the deep inspiration breath hold technique in patients with mediastinal Hodgkin’s lymphoma. Int J Radiat Oncol Biol Phys.

[85] Charpentier AM, Conrad T, Sykes J (2014). Active breathing control for patients receiving mediastinal radiation therapy for lymphoma: impact on normal tissue dose. Practical Radiation Oncology.

[86] Petersen PM, Aznar MC, Berthelsen AK (2015). Prospective phase II trial of image-guided radiotherapy in Hodgkin lymphoma: benefit of deep inspiration breath-hold. Acta Oncol.

[87] Kan MW, Leung LH, Wong W, Lam N (2008). Radiation dose from cone beam computed tomography for image-guided radiation therapy. Int J Rad Oncol Biol Phys.

[88] Islam MK, Purdie TG, Norrlinger BD, Alasti H, Moseley DJ, Sharpe MB, Siewerdsen JH, Jaffray DA (2006). Patient dose from kilovoltage cone beam computed tomography imaging in radiation therapy. Med Phys.

[89] Morin O, Gillis A, Descovich M, Chen J, Aubin M, Aubry JF, Chen H, Gottschalk AR, Xia P, Pouliot J (2007). Patient dose considerations for routine megavoltage cone-beam CT imaging. Med Phys.

[90] Dabaja BS, Hoppe BS, Plastaras JP (2018). Proton therapy for adults with mediastinal lymphomas: the international lymphoma radiation oncology group guidelines. Blood.

